# Pt Single Atoms Loaded on Thin‐Layer TiO_2_ Electrodes: Electrochemical and Photocatalytic Features

**DOI:** 10.1002/smll.202404064

**Published:** 2024-08-18

**Authors:** Xin Zhou, Yue Wang, Nikita Denisov, Hyesung Kim, Jihyeon Kim, Johannes Will, Erdmann Spiecker, Alexander Vaskevich, Patrik Schmuki

**Affiliations:** ^1^ Department of Materials Science WW4‐LKO Friedrich‐Alexander‐University of Erlangen‐Nuremberg Martensstrasse 7 91058 Erlangen Germany; ^2^ Institute of Micro‐ and Nanostructure Research & Center for Nanoanalysis and Electron Microscopy (CENEM) IZNF Friedrich‐Alexander‐Universität Erlangen‐Nürnberg Cauerstraße 3 91058 Erlangen Germany; ^3^ Department of Molecular Chemistry and Materials Science Weizmann Institute of Science Rehovot 7610001 Israel; ^4^ Regional Centre of Advanced Technologies and Materials Šlechtitelů 27 Olomouc 78371 Czech Republic

**Keywords:** anatase thin film electrode, electrocatalytic hydrogen evolution, graphene, photocatalytic hydrogen evolution, Pt Single atom

## Abstract

Recently, the use of Pt in the form of single atoms (SA) has attracted considerable attention to promote the cathodic hydrogen production reaction from water in electrochemical or photocatalytic settings. First, produce suitable electrodes by Pt SA deposition on Direct current (DC)‐sputter deposited titania (TiO_2_) layers on graphene—these electrodes allow to characterization of the electrochemical properties of Pt single atoms and their investigation in high‐resolution HAADF‐STEM. For Pt SAs loaded on TiO_2_, electrochemical H_2_ evolution shows only a very small overpotential. Concurrent with the onset of H_2_ evolution, agglomeration of the Pt SAs to clusters or nanoparticles (NPs) occurs. Potential cycling can be used to control SA agglomeration to variable‐size NPs. The electrochemical activity of the electrode is directly related to the SA surface density (up to reaching the activity level of a plain Pt sheet). In contrast, for photocatalytic H_2_ generation already a minimum SA density is sufficient to reach control by photogenerated charge carriers. In electrochemical and photocatalytic approaches a typical TOF of ≈100–150 H_2_ molecules per second per site can be reached. Overall, the work illustrates a straightforward approach for reliable electrochemical and photoelectrochemical investigations of SAs and discusses the extraction of critical electrochemical factors of Pt SAs on titania electrodes.

## Graphical abstract

An ideal electrode for single atom (SA) electrochemistry studies and concurrent microscopy is designed. Its application is shown for the hydrogen evolution reaction of Pt single atoms on thin‐film TiO_2_ as well as ongoing SA agglomeration during the reaction. Finally, the behavior is directly compared to the photocatalytic H_2_ evolution of the same platform.

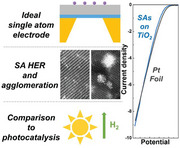

## 1 Introduction

Photocatalytic or photoelectrochemical water splitting using semiconductor materials has gained wide interest in view of sustainable hydrogen production.^[^ ^1^ ^]^ In photoelectrochemical H_2_ production, the anodic reaction, that is, the oxidation reaction H_2_O to O_2_ takes place on the semiconductor, the reduction reaction, that is, the production of H_2_ (2H^+^ + 2e^−^ → H_2_) occurs at a counter electrode, that is mostly a Pt sheet or net.^[^ ^2^, ^3^, ^4^ ^]^ In a plain photocatalytic setting, both the anodic and cathodic reactions of water splitting, take place on the same photocatalytic surface, for example on two locations of a nanoparticle suspended in an aqueous environment.^[^ ^5^, ^6^, ^7^, ^8^ ^]^ In both fields—as a photocatalyst or as a photoanode—titanium dioxide (TiO_2_) has been extensively studied due to its excellent stability, abundance, and favorable band structure for water‐splitting reactions.^[^ ^9^, ^10^, ^11^ ^]^ In the photocatalytic case, often TiO_2_ nanostructures (such as nanoparticles) are used, and as there is no additional potential applied, the nanoparticle TiO_2_ needs to be modified with Pt or other co‐catalysts to sufficiently facilitate the hydrogen evolution reaction (HER),^[^ ^12^, ^13^, ^14^, ^15^ ^]^ i.e., Pt acts as a catalyst for the transfer of electrons to water or H^+^ in the environment and aids the formation of H_2_. While Pt has been used for years in the form of nanometer sized clusters or nanoparticles deposited by various techniques on TiO_2_,^[^ ^16^, ^17^, ^18^, ^19^ ^]^ more recently the use of platinum in the form of single atoms (SAs) has attracted strong interest. This is because SAs represent maximized utilization efficiency, that is, a maximum co‐catalytic activity at a minimum of precious metal use.^[^ ^20^, ^21^, ^22^, ^23^, ^24^, ^25^, ^26^ ^]^

Platinized titania nano‐structures (such as nanoparticles,^[^ ^27^, ^28^ ^]^ nanosheets,^[^ ^14^ ^]^ nanorods,^[^ ^29^ ^]^ etc.) for photocatalytic H_2_ generation are mostly used in the form of suspensions. On such free‐floating entities, the reaction rate is a result of both the reduction of protons and the oxidation of water or a sacrificial agent (and the anodic or cathodic reaction can be rate‐controlling).^[^ ^30^ ^]^ For such suspensions, the investigation of the individual reactions is not facile and straightforward. To study the reduction and/or the oxidation contributions by electrochemical or photoelectrochemical measurements, an electrode needs to be constructed.^[^ ^31^ ^]^ Usually, the required electrodes are made from TiO_2_ powder, using processes that involve preparing a paste or slurry using the TiO_2_ powder and a suitable binder, deposit this mixture onto a conductive substrate, and then anneal the composite to an electrode.^[^ ^32^, ^33^ ^]^

Such electrodes, however, have considerable drawbacks such as homogeneity and interfacial resistance: When using TiO_2_ powder and binders, achieving a highly uniform and controlled layer thickness, porosity, surface area, and charge transport characteristics is challenging.^[^ ^34^, ^35^ ^]^ The presence of binders and the particulate nature of TiO_2_ powder can introduce additional interfacial resistance. As a result, such electrodes easily lead to erroneous electrochemical measurements and notoriously suffer from bad reproducibility.^[^ ^36^, ^37^ ^]^ All these effects are even more severe if such an electrode is decorated with nanoclusters or active single atoms (they can be buried in a binder or be placed on binder sites, etc).^[^ ^38^ ^]^ Moreover, if the goal is to study co‐catalysts in the form of SAs, powder electrodes do not allow a reliable and complete observation of individual SAs by the most direct observation tool for SA which is high‐angle annular dark‐field scanning transmission electron microscopy (HAADF‐STEM).^[^ ^39^, ^40^, ^41^, ^42^ ^]^

Therefore, in the present work, we first establish a reliable experimental platform that is based on a highly defined TiO_2_ layer sputter deposited on a conductive back contact allowing for precise control over the layer thickness and properties, including a high level of electrochemical control and importantly: TEM transparency. For this, we grow direct current (DC) sputter deposited anatase thin films on graphene and use this platform not only for photocatalytic H_2_ evolution but also for HAADF‐STEM investigations. These compact, flat layers indeed provide a simple and defined geometry for electrochemistry. In the present work, we decorate these layers with Pt SAs and investigate the electrochemical behavior, that is the HER on the Pt SA‐loaded electrodes (as well as polarization effects on the SA agglomeration surface configuration). Electrochemical examination shows that dark current flow in these TiO_2_ electrodes—as expected—only occurs when a potential negative to the flatband potential is applied. We find that the electrocatalytic hydrogen evolution is directly determined by the density of co‐catalytic sites (Pt SA density) up to maximum surface density of ≈5.5 × 10^6^ µm^−2^—here the Pt SA decorated electrode provides an activity that is comparable to a plain Pt sheet. However, for such a Pt SA decorated electrode, concurrent with the onset of H_2_ evolution, there is significant agglomeration of the SAs to nanoparticles (NPs) observable that accordingly lowers the electrode activity. This agglomeration of SAs to various‐size nanoparticles can be controlled by voltage cycling. Moreover, we compare the data to the case of using Pt SAs for photocatalytic HER and in contrast to electrochemical HER, the photocatalytic HER activity becomes saturated at very low Pt SA loading (in the photocatalytic case the limit of activity is typically established by light and carrier harvesting, rather than by a limiting number of co‐catalytic sites.

Overall, combined photocatalytic and electrochemical results demonstrate the versatility of the here introduced platform of sputter‐deposited layers on graphene for electrochemistry, photoelectrochemistry, and the atomic morphology of SA‐decorated semiconductor surfaces, and the present investigations give an example of most direct use of the electrode for extracting SA‐related electrochemical data for photocatalysis.

## 2 Results and Discussion

**Figure 1:**
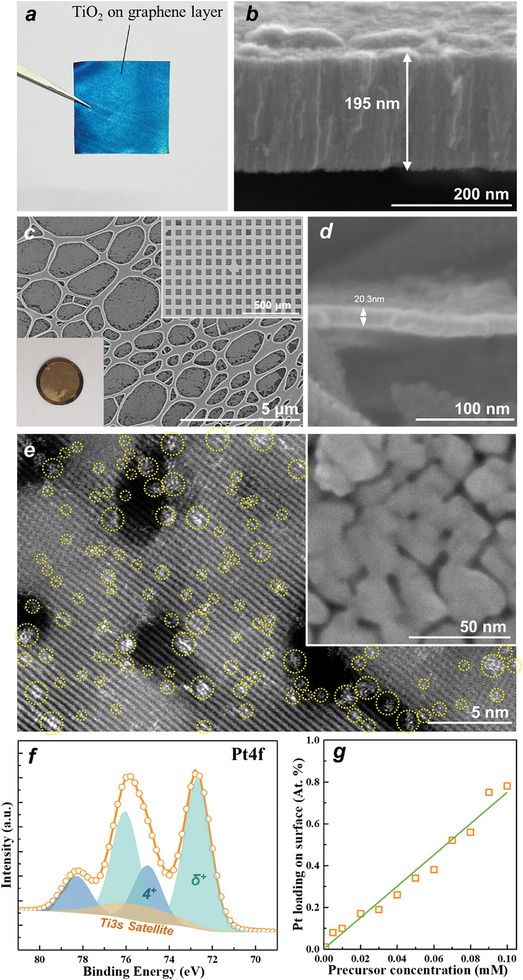
a) Optical picture of 200 nm sputtered titania layer on graphene sheets; b) SEM image of the cross‐section of the sputtered titania layer on graphene sheets; c) SEM image of the top view of sputtered titania layer on TEM membrane (the insets show an optical image and a lower magnification of SEM image); d) SEM image of the cross‐section of the 20 nm sputtered titania layer on TEM grid; e) HAADF‐STEM images of Pt SA‐loaded TiO_2_ layers (the inset shows the SEM image of Pt SA‐loaded TiO_2_ layers); f) XPS Pt4f spectra of Pt SA‐loaded TiO_2_ layers; g) Atomic concentration of Pt deposition on the TiO_2_ layer under different Pt precursor concentration treatments.

In a first step, we produced sputter deposited titania layers on graphene sheets as described in Experimental Section and as illustrated in **Figure** **1** (see optical image in Figure 1a and SEM image in Figure 1b). Layers for electrochemistry were sputtered on graphene sheets to a TiO_2_ thickness of ≈200 nm (Figure 1b), for TEM investigation, a layer with a thickness of 20 nm was deposited on TEM supports (Figure 1c,d). All the layers were annealed in air at 450 °C and consisted of anatase phase TiO_2_ as evident from Raman spectroscopy measurements (Figure S1, Supporting Information).

Then we deposited Pt SAs onto the titania layers by a previously described “reactive” approach.^[^ ^43^ ^]^ Previous work in detail characterized this deposition approach using a broad range of techniques.^[^ ^22^, ^23^, ^24^, ^26^, ^44^ ^]^ Figure 1e shows a HAADF‐STEM image taken from such an anatase TiO_2_ thin layer on the TEM grid. In this case, the anatase TiO_2_ layer was exposed for the reactive deposition to a 2 mM H_2_PtCl_6_ solution for 1 h. In Figure 1e, Pt atoms are marked with yellow circles, and from a statistical evaluation a Pt SA density of ≈1.8 × 10^6^ µm^−2^ can be determined. As expected, in the high‐resolution SEM images of this layer (Figure 1e inset), no distinct clusters or nanoparticles can be seen after SA deposition as the Pt feature sizes are clearly below the resolution of the high‐resolution SEM. For such a layer the XPS spectrum of the Pt 4f region (Figure 1f) shows a typical 4f doublet with a clear Pt 4f_7/2_ peak at a position of 72.4 eV. This is fully in line with previous work and typical for SA Pt coordinated to oxygen on a TiO_2_ surface with a formal charge of δ ≈+2 on Pt^δ^.^[^ ^24^, ^26^, ^43^, ^45^ ^]^ With increasing precursor concentration, the Pt loading (determined from XPS) increases, as shown in Figure 1g. Using the correlation of HAADF‐STEM and XPS allows for estimating the SA densities given in Table S1 (Supporting Information), that is, we may establish SA densities in the range of ≈10^4^–10^6^ µm^−2^.

**Figure 2:**
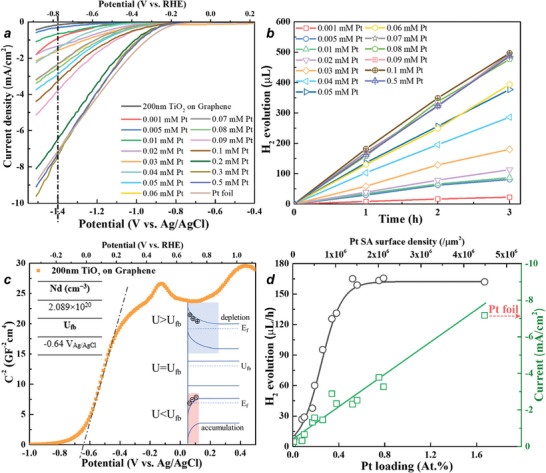
a) LSV curves of Pt‐loaded TiO_2_ layers at different Pt precursor concentrations, bare TiO_2_ layers, and Pt foil; b) Photocatalytic H_2_ evolution of Pt‐loaded TiO_2_ layers at different Pt precursor concentrations; c) Mott–Schottky plot of 200 nm sputtered titania layer on graphene sheets (the inset illustrates schematic band diagrams for an n‐type semiconductor/liquid electrolyte interface); d) Photocatalytic H_2_ evolution rate and current density (at −1.4 V vs Ag/AgCl) at different Pt loadings on the TiO_2_ surface.

We then used this series of samples with different loading densities of Pt SAs to acquire linear sweep voltammetry (LSV) measurements as shown in **Figure** **2**a and we evaluated the photocatalytic H_2_ production for these layers (Figure 2b). From the electrochemical data in Figure 2a it can be seen, compared to bare TiO_2_, all Pt SA decorated samples show a clear onset of cathodic currents at ≈−0.8 to −1.0 V_Ag/AgCl_ with a potential for 1 mA of E_1 mA_  =  −1.49 to −0.98 V_Ag/AgCl_. Figure S2 (Supporting Information) shows a compilation of the data for the differently loaded electrodes. If we consider E_1 mA_ ≈−0.95 V_Ag/AgCl_ for Pt foil, one can see that the onset of H_2_ evolution for Pt SA decorated electrode with ≈0.5 at.% Pt (1.4 × 10^6^ SAs µm^−2^, extrapolated from Figure S2, Supporting Information) shows an overpotential of ≈250 mV compared to a bulk Pt electrode, whereas higher Pt SA densities, such as 5.5 × 10^6^ µm^−2^ show only a very minor (≈30 mV) overpotential. That is, for a high loading of Pt SAs on TiO_2_ under the present conditions there is no significant influence of the TiO_2_ substrate to the HER overpotential.

Note that an n‐type semiconductor such as TiO_2_ (in the dark) is only a viable electrode once the semiconductor–electrolyte interface is polarized to a current passing state—, that is, a potential that is negative to the flat‐band potential U_fb_ (as illustrated in the inset of Figure 2c). To determine U_fb_, we acquired Mott–Schottky type of measurements (capacitance vs voltage characteristics C^−2^ vs U) of the plain TiO_2_ electrode (Figure 2c). The extrapolation of the linear part of the Mott–Schottky plot yields a flat band potential of ≈−0.6 V (Ag/AgCl) which is in good agreement with the literature for TiO_2_ in neutral electrolytes.^[^ ^46^, ^47^, ^48^, ^49^ ^]^ Additionally, a similar value for the flat‐band potential (U≈ −600 mV) is obtained by light saturation measurements (shown in Figure S3, Supporting Information)., that is, the onset of H_2_ evolution in Figure 2a in every case is negative to U_fb_.

Regarding the magnitude of the electrochemical HER, the data in Figure 2a shows that the higher the precursor Pt loading (Table S1, Supporting Information), the higher the overall hydrogen evolution current density. If we evaluate the current density at a fixed potential (here −1.4 V _Ag/AgCl_), then we obtain a steady increase with Pt loading (Figure 2d). This nearly linear dependence of the H_2_ production rate on the Pt loading shows that the hydrogen production current density scales very well with the surface density of Pt SA. That is, the Pt loading and thus in a first approximation the number of active sites (SA density) determines current flow, i.e., the H_2_ production rate—this holds up to the point where the activity reaches the activity of a metallic Pt sheet.

Based on the H_2_ evolution activity and the Pt‐SA density, we calculated turnover frequency (TOF) versus voltage plots for the various electrodes (see Figure S4a, Supporting Information). Evidently, higher‐loaded electrodes show a lower TOF while lower‐loaded electrodes seem to reach a maximum of ≈100–150 at a voltage of −1.5 V_Ag/AgCl_ (Figure S4b, Supporting Information).

This is roughly in line with previous estimations of the maximum TOF on Pt SAs^[^ ^24^, ^25^ ^]^ or Pt NPs.^[^ ^50^ ^]^ Most remarkable is that the decoration with sufficient SAs is as good as a plain Pt sheet, that is, this is much more efficient in terms of Pt utilization (under our conditions) than the use of a plain Pt electrode.

In a wider context, it is interesting to compare the electrochemical data with the photocatalytic H_2_ evolution activity of the same SA‐loaded TiO_2_ layers in Figure 2b (measured using illumination with a UV LED (365 nm, 65 mW cm^−2^) and compared using an AM 1.5 solar simulator for comparison (100 mW cm^−2^; Figure S7, Supporting Information)—see ref. [24] for details to the experiment. For all the differently Pt SA loaded samples, we obtain a linear increase of the photocatalytic production over illumination time, therefore from the slope of the data, the reaction rate can easily be evaluated. In Figure 2d, these rates are plotted against the Pt loading of the surface. Notably, with a higher loading the activity increases drastically but already at a very low Pt loading of 0.5 at.% Pt, that is, a Pt SA density of ≈1.4 × 10^6^ SA Pt  µm^−2^, a saturation of the activity occurs.

The fact that for photocatalytic HER a saturation of activity is observed at low Pt loading, while for electrochemical HER no such limit could be observed (up to a maximum loading condition that is comparable to a plain Pt electrode) is noteworthy. The result can be ascribed to the fact that under our illumination/semiconductor‐conditions, light harvesting from the semiconductor provides a much lower interfacial electron flux toward the catalytic SAs than in a typical electrochemical experiment. Our illumination conditions (we use a UV photon flux of 1.2 × 10^21^ s^−1^ m^−2^ corresponding to ≈10 suns) convert to a maximum current density of 1–2 mA cm^−2^. This means that in photocatalysis already at a very small amount of Pt SA loading, the co‐catalytic activity is sufficient to “deal” efficiently with all charge carriers arriving at an active site.

**Figure 3:**
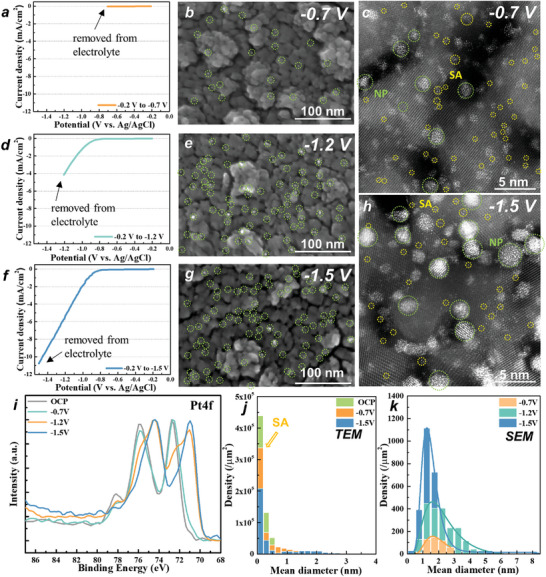
a,d,f) LSV curves of 2 mm Pt loaded TiO_2_ layer with different end potentials (−0.7, −1.2, and −1.5 V vs Ag/AgCl); b,e,g) SEM images of electrodes after exposure to different potentials (−0.7, −1.2, and −1.5 V vs Ag/AgCl); c,h) TEM images of electrodes after exposure to different potentials (−0.7 and −1.5 V vs Ag/AgCl); i) XPS Pt4f spectra of Pt loaded TiO_2_ layers (before immersion (OCP) and after polarization (−0.7, −1.2, and −1.5 V vs Ag/AgCl)); j,k) Statistical evaluation of Pt SAs and Pt NPs from TEM and SEM images with different polarization potential.

In the context of using SAs as catalysts, a general question is always after the potential agglomeration of the SAs during reaction. While we recently discussed this point for photocatalytic experiments in detail,^[^ ^26^ ^]^ we will briefly touch on it here for electrochemical experiments. For this, we acquired potential sweeps to different end potentials and then removed the electrodes from the electrolyte. **Figure** **3**b–h shows SEM and HAADF‐STEM images of electrodes after exposure to different potentials, i.e., i) removal directly after immersion (Figure 1e) and after polarization ii) to −0.7, iii) to −1.2, and iv) to −1.5 V versus Ag/AgCl. At low cathodic potentials, such as −0.7 V, mild agglomeration of the single atoms occurs, with a density of 4.6 × 10^2^ NPs µm^−2^. Only at potentials that correspond to values clearly beyond the H_2_ onset potential, in our case taken at −1.2 V (Figure 3e) and −1.5 V (Figure 3g), SEM reveals strong agglomeration—the more cathodic, the more pronounced the agglomeration and the formation of nanoparticles. At −1.5 V, the density of Pt nanoparticles increases to 2.8 × 10^3^ NPs µm^−^^2^, and these nanoparticles agglomerate into larger clusters, with diameters reaching several nanometers.

Figure 3j,k and Figure S8 (Supporting Information) show the statistical evaluation of the Pt size distribution for different voltages and the resulting particle size increase. XPS (Figure 3i) for these samples show that at higher cathodic voltages a clear shift of the XPS Pt 4f_7/2_ peak from 72.4 to 70.4 eV which corresponds to an increasing conversion to metallic Pt^0^ well in line with the observed particle formation in SEM.^[^ ^26^, ^45^ ^]^ A quantitative evaluation of the XPS data yields at −0.7 V a ≈5% increase of the metallic Pt^0^ and reaches 87% at −1.5 V (Figure S9 and Table S2, Supporting Information).

The fact that at voltages negative to the onset potential of H_2_ significant agglomeration can be observed is very well in line with a previous DFT‐model^[^ ^26^ ^]^ for photocatalytic H_2_ production on Pt SAs. It showed that the key factor for Pt agglomeration is the formation of the intermediate Pt–H species which leads to a weakening of the Pt─substrate bonds. (This implies that the Pt–H species can become mobile on the TiO_2_ substrate and agglomerate to Pt^0^ particles).

**Figure 4:**
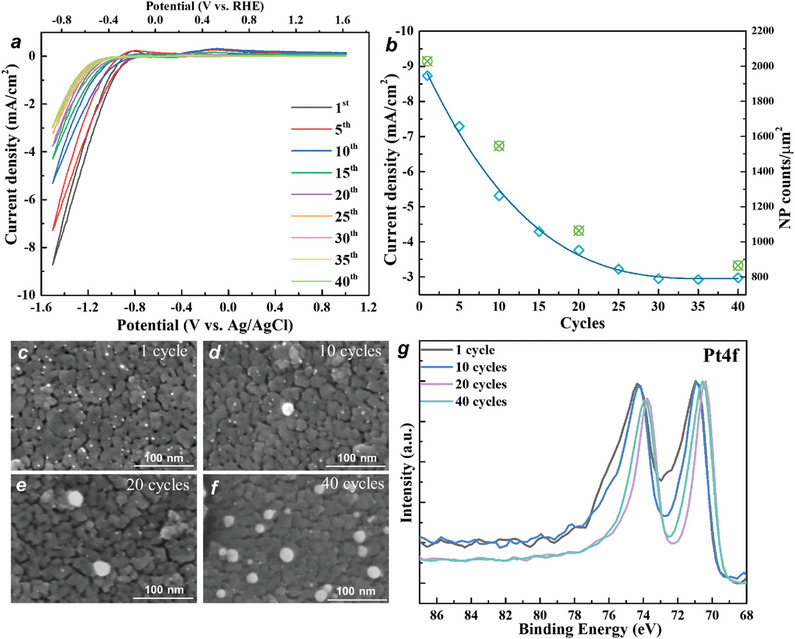
a) CV curves of 2 mm Pt loaded TiO_2_ layer at a scan rate of 5 mV s^‐1^; b) Current density of Pt loaded TiO_2_ layer at −1.5 V for different cycles and statistical evaluation of Pt NPs from SEM images; c–f) SEM images Pt deposited layers after different cycles; g) XPS Pt4f spectra of Pt loaded TiO_2_ layers after different cycles.

In order to elucidate the effect of agglomeration on the electrochemical H_2_ evolution activity we carried out repeated scans, i.e., cyclic voltammograms (CVs) as shown in **Figure** **4**a. Evidently with an increasing scan number the electrode gets increasingly deactivated (Figure 4b). At the same time, SEM in Figure 4c–f shows an increase in Pt particle sizes on the electrode surface. For 1 cycle, SEM visible Pt particles have an average diameter of ≈ 2.5 nm, for 10 cycles ≈ 3.0 nm, for 20 cycles ≈ 3.8 nm, and for 40 cycles ≈ 7.5 nm. From the XPS peak deconvolution of the Pt 4f peak, we see an increasing contribution of metallic Pt^0^ (Pt 4f_7/2_ ≈ 70.4 eV) and a dropping contribution of Pt^δ+^ (Pt 4f_7/2_ ≈ 72.7 eV). From Figure 4b we observe a deactivation (a drop in the peak current) from ≈9 to 3 mA cm^−2^ after 40 electrochemical cycles. Interestingly, if we evaluate the Pt active sites (SEM visible nanoparticles), then we observe also a drop of a factor of 3, this irrespective of the average size of the particles., that is, the main detrimental effect is in the loss of active sites while the particle size seems to hardly matter.

In summary, the work shows how to construct a reliable and efficient electrode for the characterization of Pt SAs electrochemistry and photoelectrochemistry. For electrochemical HER, using a high density of Pt SAs allows to reach high HER current densities. In fact, at a density of ≈5.5 × 10^6^ SA µm^−2^ we reach the same HER current density as metallic Pt—this means a much better utilization of SA Pt for electron transfer than in Pt sheets. In comparison, in photocatalytic HER, even under ≈10x terrestrial illumination conditions (65 mW cm^−2^ UV), a much lower interfacial carrier flux is typically achieved—this accordingly leads to a lower required density of Pt sites in photocatalysis than for typical electrocatalytic conditions. Agglomeration of SAs during HER is common to photocatalysis and electrocatalysis—it can be ascribed to same the destabilizing effect (formation of mobile Pt–H intermediates on TiO_2_).^[^ ^26^ ^]^

## 3 Conclusion

In this work, we evaluate the electro‐ and photocatalytic activity of Pt SAs on TiO_2_ electrodes fabricated by sputter‐deposition on a graphene back‐contact. These electrodes allow for a reliable and direct investigation of electrochemical properties as well as SA characterization by HAADF‐STEM. For these electrodes, dark current can only flow when the applied potential is more negative than the flatband potential, enabling electron injection into the TiO_2_ conduction band and subsequent interfacial charge transfer processes.

Pt SAs on the anatase titania surface catalyze the electrochemical HER. The activity of the electrode increases linearly with loading up to a density of ≈5.5 × 10^6^ SA µm^−2^—at this density, the activity of plain Pt sheet is reached (i.e., showing similar HER current densities, onset, and overpotential).

For photocatalysis, using ≈10 sun irradiation, photoinduced currents in the 1 mA range can be reached and accordingly already a density of 1.4 × 10^6^ SA Pt µm^−2^ is fully sufficient to achieve a maximized co‐catalytic activity. This behavior can be ascribed to the fact that in such photocatalytic light harvesting, only a much lower electron flux (converted light flux) can be usually achieved than under typical electrochemical conditions.

Moreover, we find that electrochemical HER using Pt SAs (very similar as reported previously for photocatalytic HER)^[^ ^26^ ^]^ leads to agglomeration of SAs to nanoparticles which lowers the effectiveness of the electrodes—the mechanism seems to be in both cases based on the formation of mobile Pt–H species.^[^ ^26^ ^]^

From an experimental viewpoint: By evaluating the electrochemistry and catalytic activity, charge transfer kinetics, stability, and dark current behavior of Pt single atoms that are loaded on well‐defined (sputter‐deposited) TiO_2_ layers, a much more accurate understanding of their performance can be achieved than using suspended particles and particle agglomerated electrodes.

## 4 Experimental Section

### Fabrication of TiO_2_ Layer

TiO_2_ layers were deposited on graphene substrates through DC magnetron sputtering (SP‐P‐US‐6M‐32 CreaTec Fischer & Co. GmbH), following the procedures previously outlined in the literature.^[^ ^51^ ^]^ Amorphous TiO_2_ layers were sputtered in an Ar/O_2_ atmosphere (6.7 × 10^−3^ mbar; volume ratio Ar:O_2_ = 10:5) using a 5″ Ti (99.995%, HMW Hauner GmbH & Co. KG) target at 500 W for 4 h, resulting in a thickness of 200 nm. Subsequently, the coated substrates were annealed at 450 °C for 1 h in air to induce crystallization of the TiO_2_ layers.

For transmission electron microscopy studies, 20 nm thin TiO_2_ layers were directly sputtered onto TEM supports (Ultrathin C Film on Lacey Carbon Support Film, Au, TED PELLA, INC.) via DC magnetron sputtering at 500 W for 30 min, followed by annealing at 450 °C in air for 1 h.

### Pt Single Atoms Decoration (Dark Deposition)

Pt SAs were loaded on the surface of TiO_2_ supports by a facile dark deposition/impregnation method. TiO_2_ layers were immersed in 10 mL of an aqueous solution of H_2_PtCl_6_·6H_2_O for 1 h. The Pt‐decorated TiO_2_ layers were washed with deionized water and dried in N_2_ gas flow.

### Characterization

The surface morphology of the samples was examined through a field‐emission scanning electron microscope (FE‐SEM, S‐4800, Hitachi). The elemental composition and chemical state of the samples were analyzed by X‐ray photoelectron spectroscopy (XPS, PHI 5600). XPS spectra were shifted to a standard Ti 2p binding energy in anatase of 458.5 eV, and the peak deconvolution was conducted using MultiPak software. High‐angle annular dark‐field scanning transmission electron microscopy (HAADF‐STEM) images of the samples were captured using a probe‐corrected scanning transmission electron microscope (Thermo Fisher Scientific Spectra 200). Raman spectra were recorded for Raman shifts between 100 and 700 cm^−1^ using a confocal Raman spectrometer (LabRAM HR Evolution, Horiba, Dresden, Germany). The 532 nm laser was used as the excitation source. The laser beam was focused on the sample surface with a ten‐fold objective. The hole and slit were fully opened at 1200 µm to record the total signal intensity over the sample bulk. The grating was set at 300 gr per mm.

### Linear Sweep Voltammetry (LSV) and Cyclic Voltammetry (CV)

Linear sweep voltammetry studies were performed in a three‐electrode electrochemical setup using a platinum plate as a counter electrode, and an Ag/AgCl (3 m KCl) reference electrode. Polarization was carried out in the dark in 0.1 m Na_2_SO_4_ aqueous solutions by sweeping the potential in the cathodic direction from −0.2 to −1.5 V (vs Ag/AgCl) with a scan rate of 5 mV s^−1^ using Autolab PG‐STAT302N potentiostat/galvanostat. Cyclic voltammetry measurements were performed in the same system and the potential was cycled between 1.0 and −1.5 V with a scan rate of 5 mV s^−1^. For reversible hydrogen electrode (RHE) conversion, the following formula was used:

(1) $$E_{\mathrm{RHE}}=E_{\mathrm{Ag}/\mathrm{AgCl}}\;+0.0591\times pH+E_{\mathrm{Ag}/\mathrm{AgCl}}^{0}$$

where $E_{\mathrm{Ag}/\mathrm{AgCl}}^{0}$ is the standard potential of the Ag/AgCl reference electrode (3 m KCl), typically 0.210 V at 25 °C.

### Photocatalytic H_2_ Evolution

The photocatalytic performance of Pt‐decorated TiO_2_ layers was evaluated under UV irradiation using methanol as a hole scavenger. The samples were placed in a quartz reactor containing a 50 vol.% methanol aqueous solution (10 mL), purged with argon for 15 min, and irradiated using either an LED (𝜆 = 365 nm, power density =  65 mW cm^−2^, exposure area = 0.785 cm^2^) or an AM 1.5G solar simulator (100 mW cm^−2^). The amount of H_2_ produced within specific time intervals was determined using a gas chromatograph (GCMS‐QO2010SE, SHIMADZU) with a thermal conductivity detector (TCD).

## Conflict of Interest

The authors declare no conflict of interest.

## Author Contributions

X.Z. and Y.W. contributed equally to this work. X.Z. and P.S. conceived the study. Y.W. conducted the primary experiments, including the synthesis and characterization of the materials. H.K. investigated the sputtered TiO₂ layers, while J.W. and E.S. carried out the microscopy analysis (HAADF‐STEM). J.K. assisted in the electrochemical experiments, and A.V. contributed to the analysis of the electrochemical data. P.S. supervised the project and provided guidance on the experimental design and data analysis. X.Z., N.D., and P.S. wrote the manuscript with input from all authors. All authors contributed to the discussion and interpretation of the data presented in this work.

## Supporting information

Supporting Information

## Data Availability

The data that support the findings of this study are available from the corresponding author upon reasonable request.
