# Supplementary material for: Pt Single Atoms Loaded on Thin‐Layer TiO2 Electrodes: Electrochemical and Photocatalytic Features
Source: Small. 2024 Aug 18;20(47):2404064. doi: 10.1002/smll.202404064 (PMC11579980; doi:10.1002/smll.202404064)
Supplement: Supplementary file 1 — Supporting Information [file SMLL-20-2404064-s001.docx]

Supporting Information

**Pt single atoms loaded on thin-layer TiO_2_ electrodes: Electrochemical and photocatalytic features**

Xin Zhou^1+^, Yue Wang^1+^, Nikita Denisov^1^, Hyesung Kim^1^, Jihyeon Kim^1^, Johannes Will^2^, Erdmann Spiecker^2^, Alexander Vaskevich^3^, Patrik Schmuki^1,4^*

^1^Department of Materials Science WW4-LKO, Friedrich-Alexander-University of Erlangen-Nuremberg, Martensstrasse 7, 91058 Erlangen, Germany

^2^Institute of Micro- and Nanostructure Research & Center for Nanoanalysis and Electron Microscopy (CENEM) IZNF, Friedrich-Alexander-Universität Erlangen-Nürnberg, Cauerstraße 3, 91058 Erlangen, Germany

^3^Department of Molecular Chemistry and Materials Science, Weizmann Institute of Science, Rehovot 7610001, Israel

^4^Regional Centre of Advanced Technologies and Materials, Šlechtitelů 27, 78371 Olomouc, Czech Republic

*Corresponding author. E-mail: [schmuki@ww.uni-erlangen.de](mailto:schmuki@ww.uni-erlangen.de)


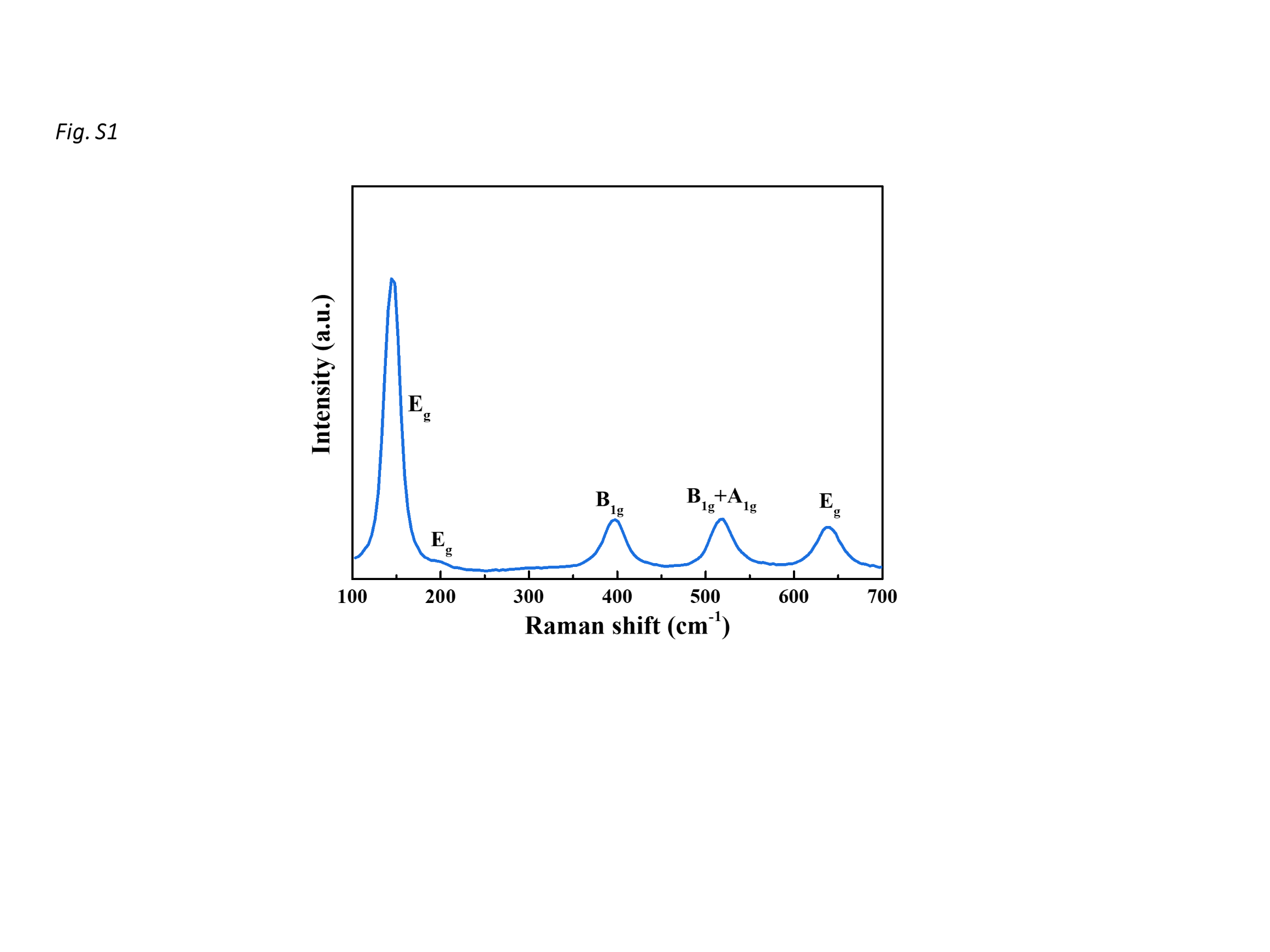


**Figure S1.** Raman spectrum of sputtered titania layer on graphene sheets after annealing.


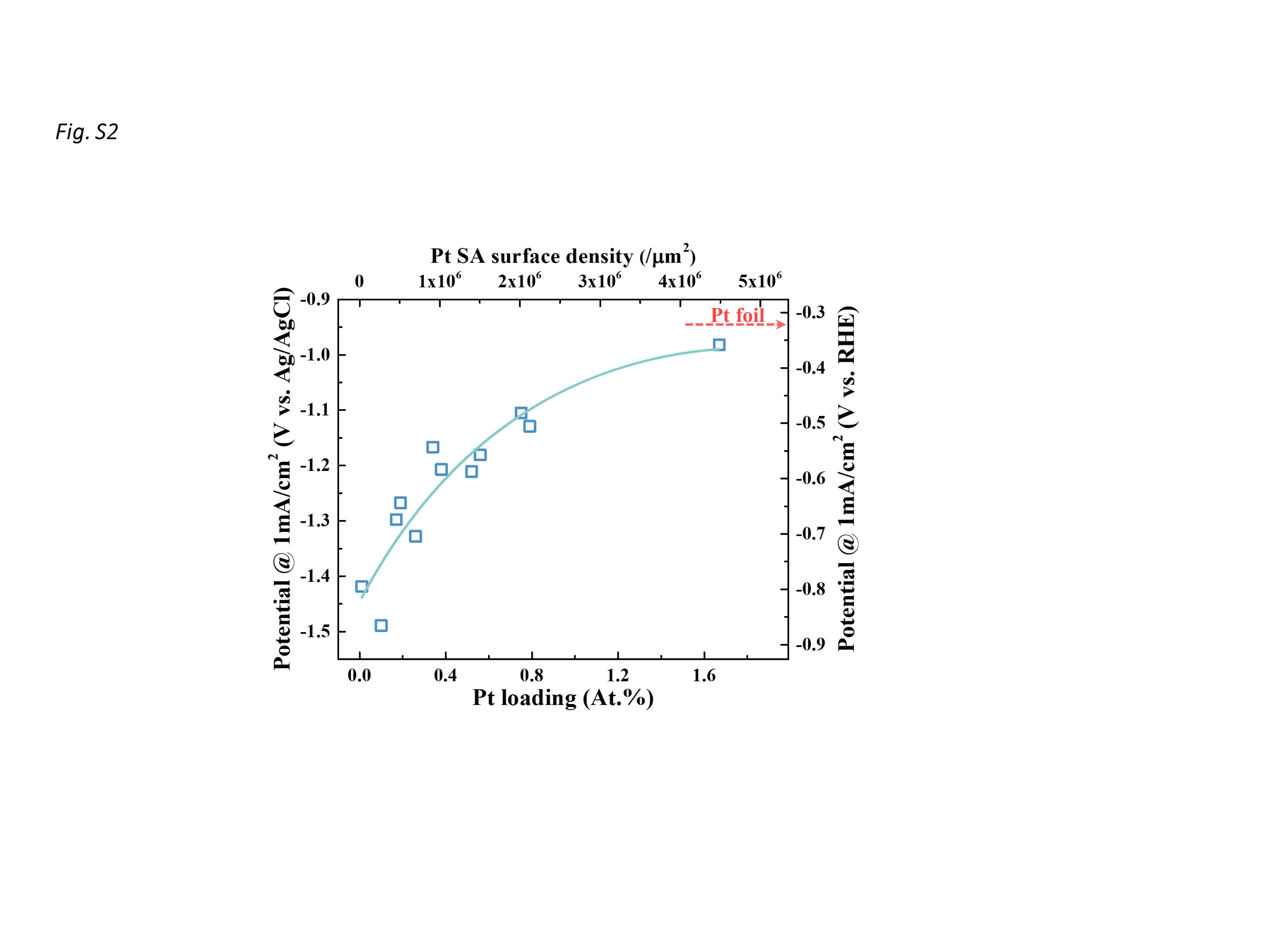


**Figure S2.** Potential at 1mA/cm^2^ of different Pt amount loaded TiO_2_ layer compared with Pt foil.


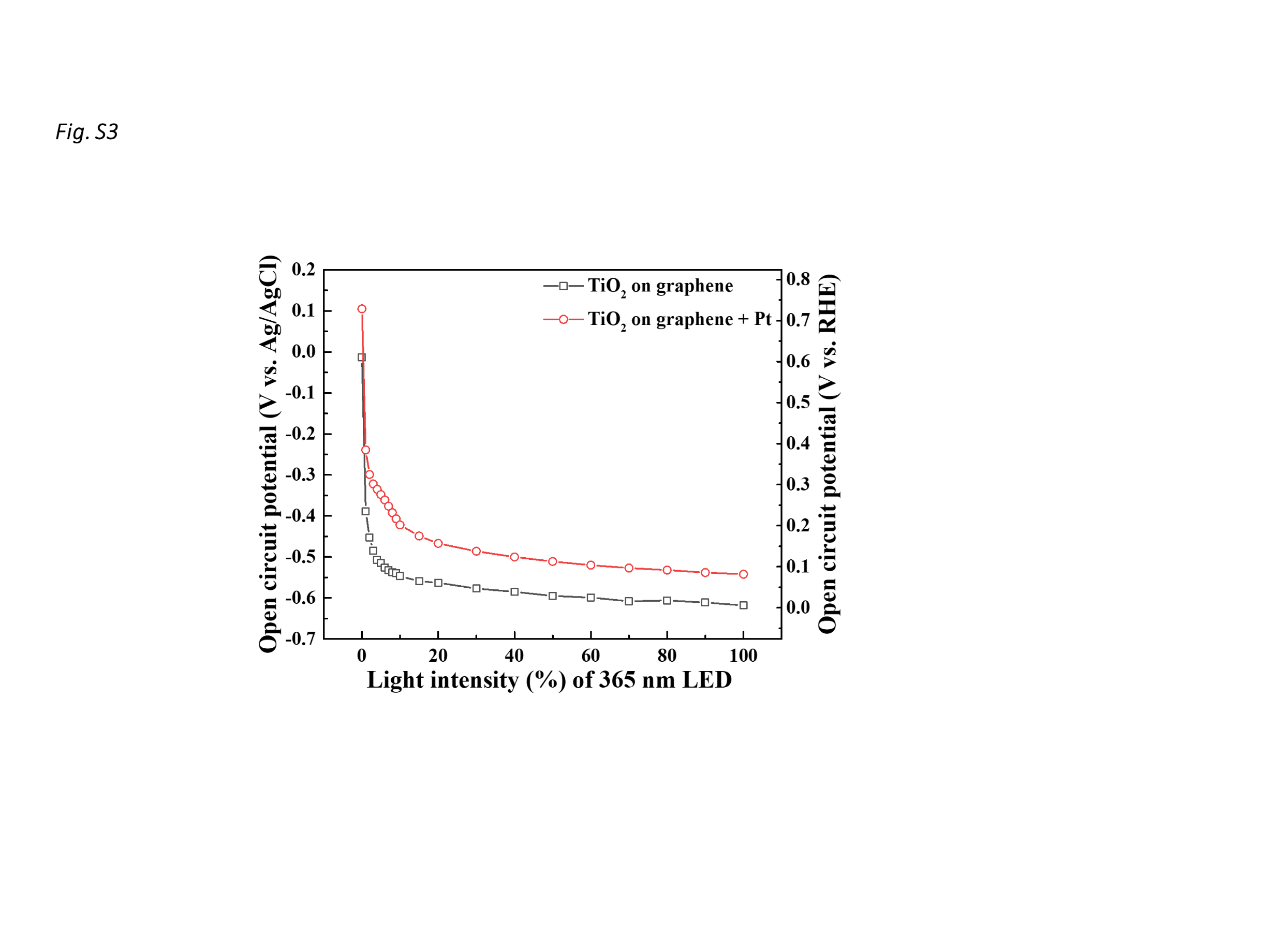


**Figure S3.** Open circuit potential of bare TiO_2_ layer and Pt SAs loaded TiO_2_ layer at different light intensity.

Determination of flat-band potential by light saturation experiments.

Here light-induced flattening of the band is generated and the resulting open circuit potential (ca. -0.5 to -0.6 V Ag/AgCl for the Pt free and the Pt loaded sample) then corresponds approximately to the respective flatband potential^[1]^.


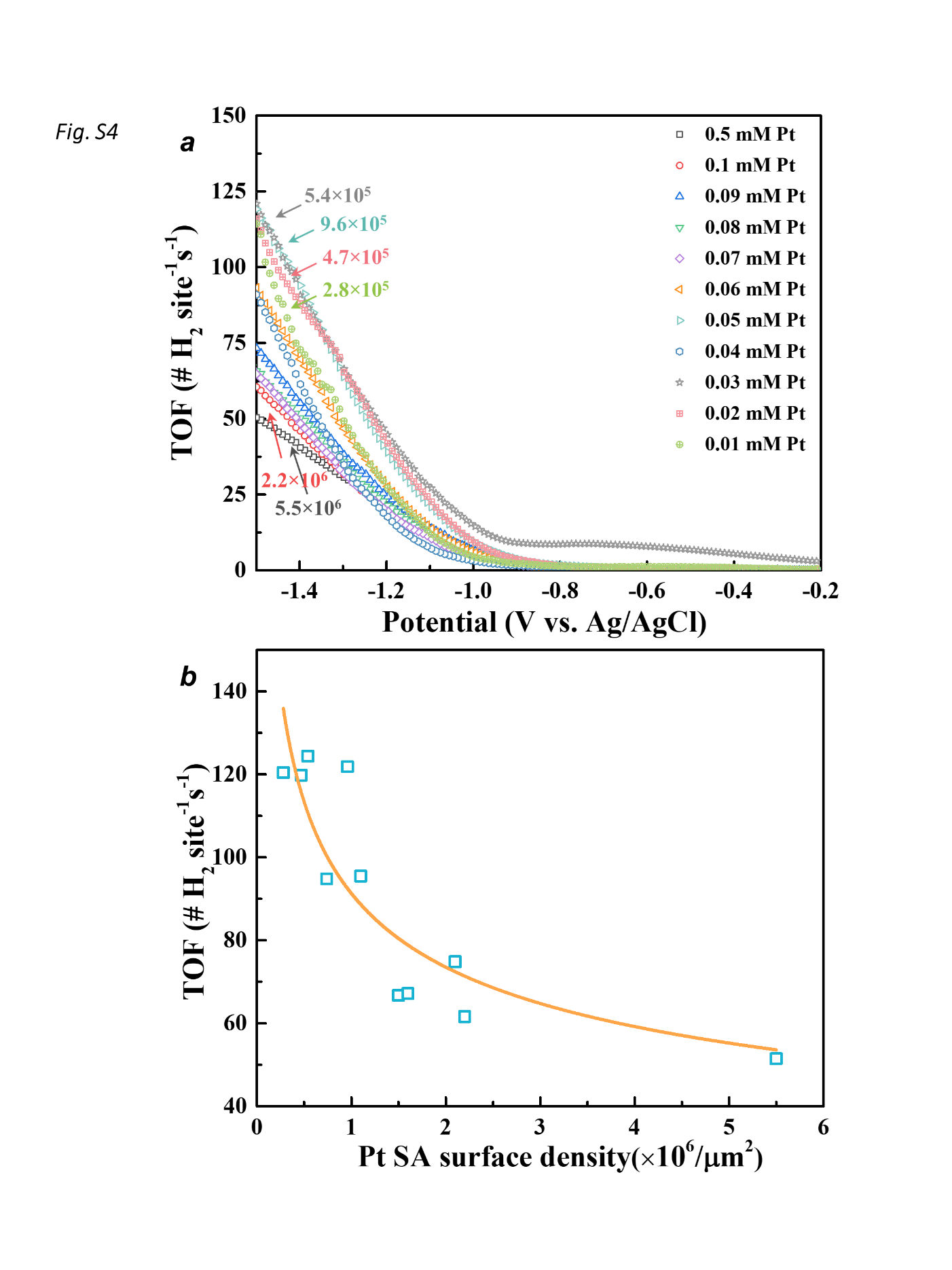


**Figure S4.** a) TOF as a function of applied bias for H_2_ electrocatalytic evolution reaction at Pt SA sites in Pt dark-deposited TiO_2_ layer; b) TOF values for different Pt SA surface density on TiO_2_ layer at -1.5V vs. Ag/AgCl.


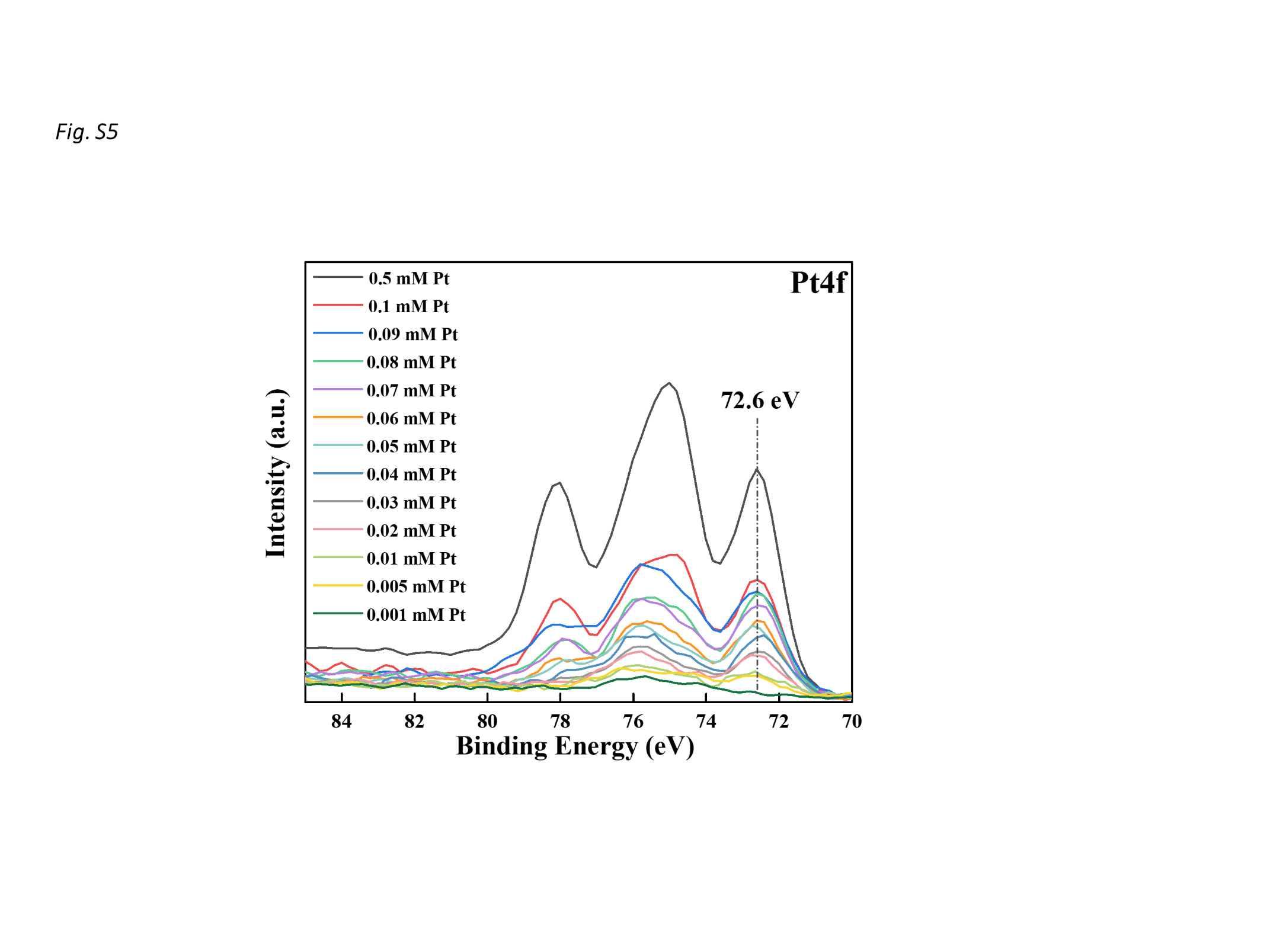


**Figure S5.** XPS Pt4f spectra of TiO_2_ layers decorated with different Pt precursor concentration.


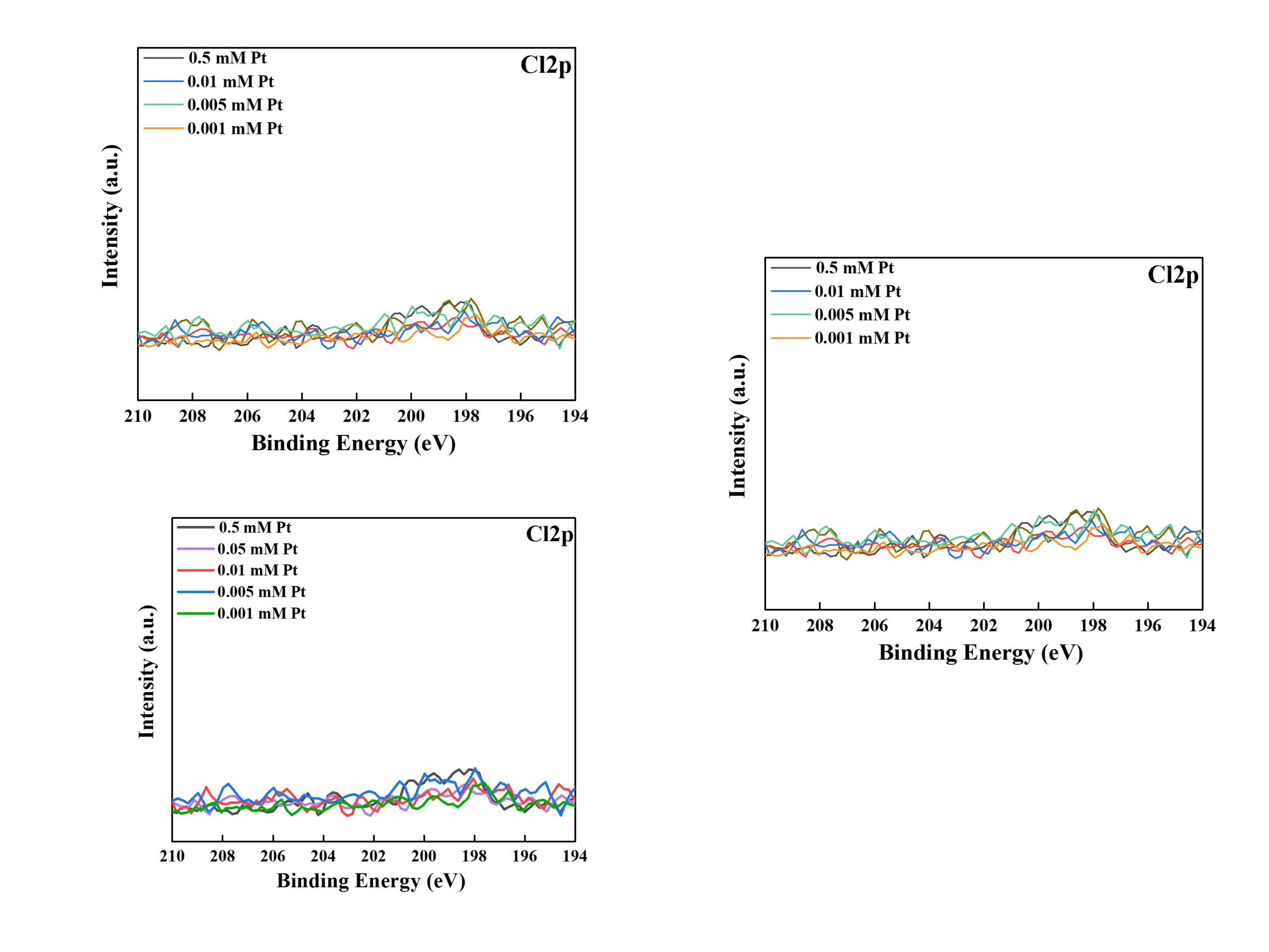


**Figure S6.** XPS Cl2p spectra of TiO_2_ layers decorated with different Pt precursor concentration.


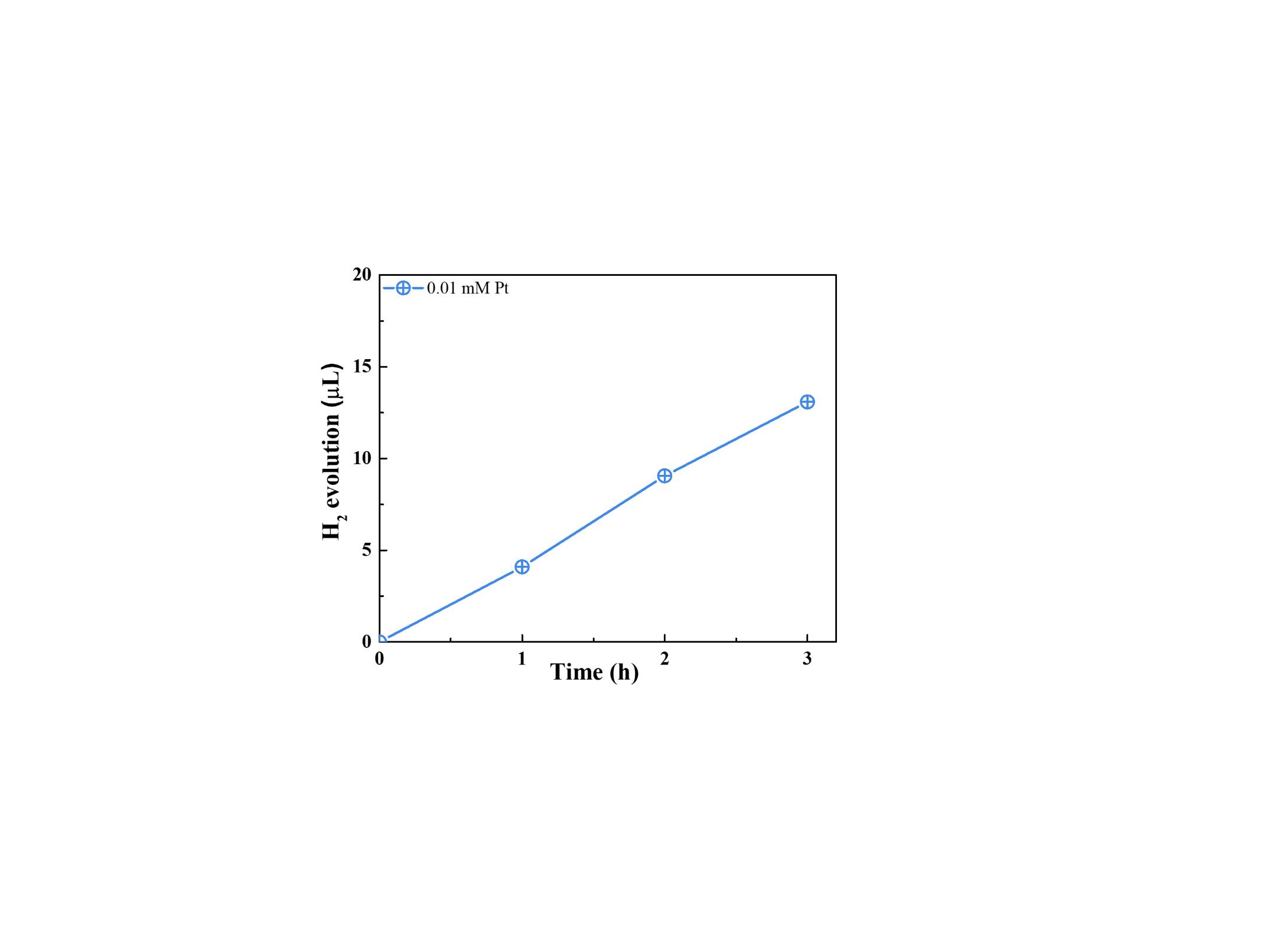


**Figure S7.** Photocatalytic H_2_ evolution of Pt loaded TiO_2_ layers under AM 1.5G solar simulator (100 mW·cm^−2^).

**Figure S8.** Area fraction of Pt single-atoms and clusters from the HAADF-STEM images of Pt SA-loaded TiO_2_ layers before and after polarization. The corresponding quantitative evaluation is provided in Fig. 3j.


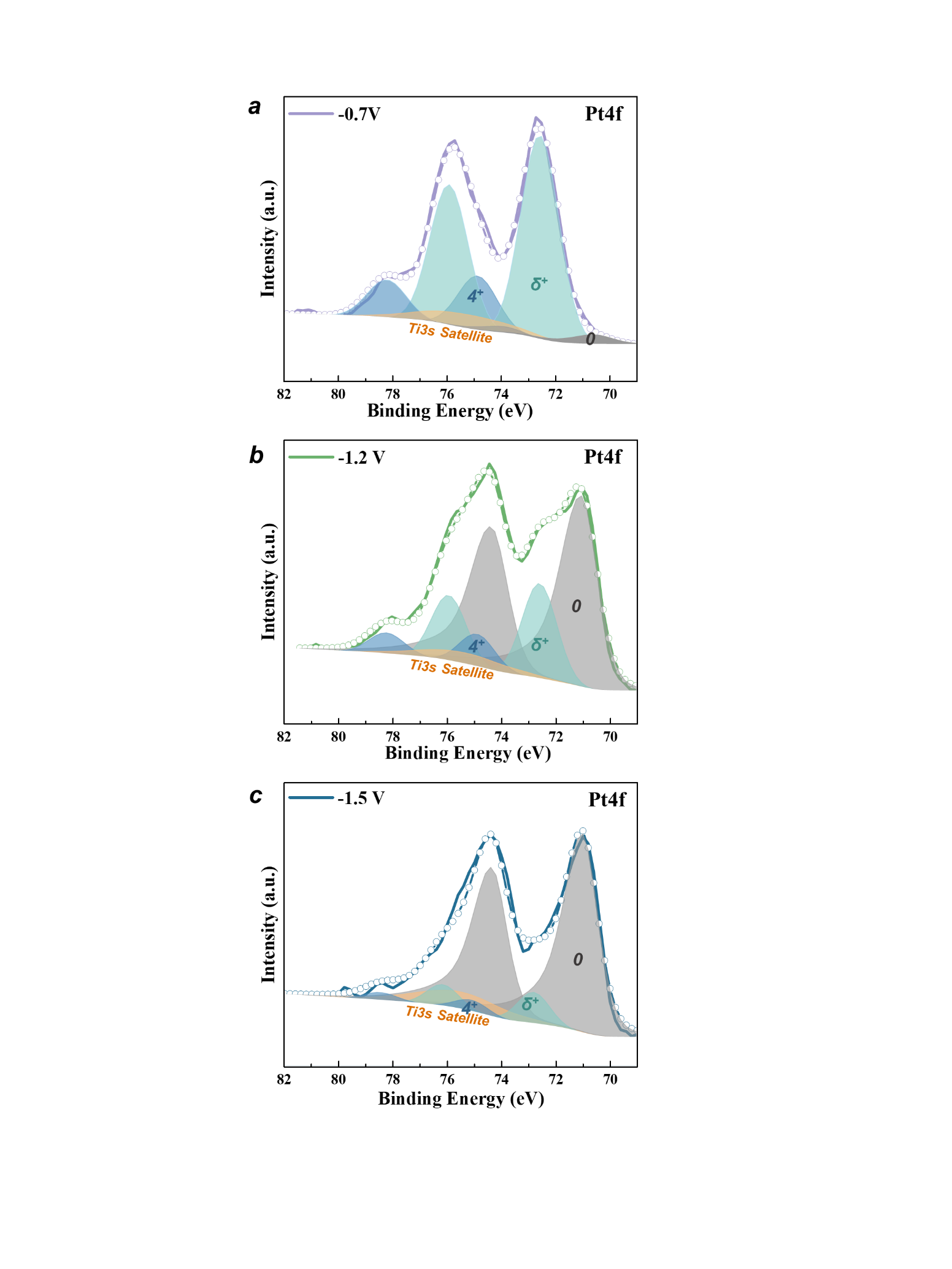


**Figure S9.** Deconvoluted XPS Pt4f spectra of Pt loaded TiO_2_ layers after polarization (-0.7V, -1.2V and -1.5V vs. Ag/AgCl)).


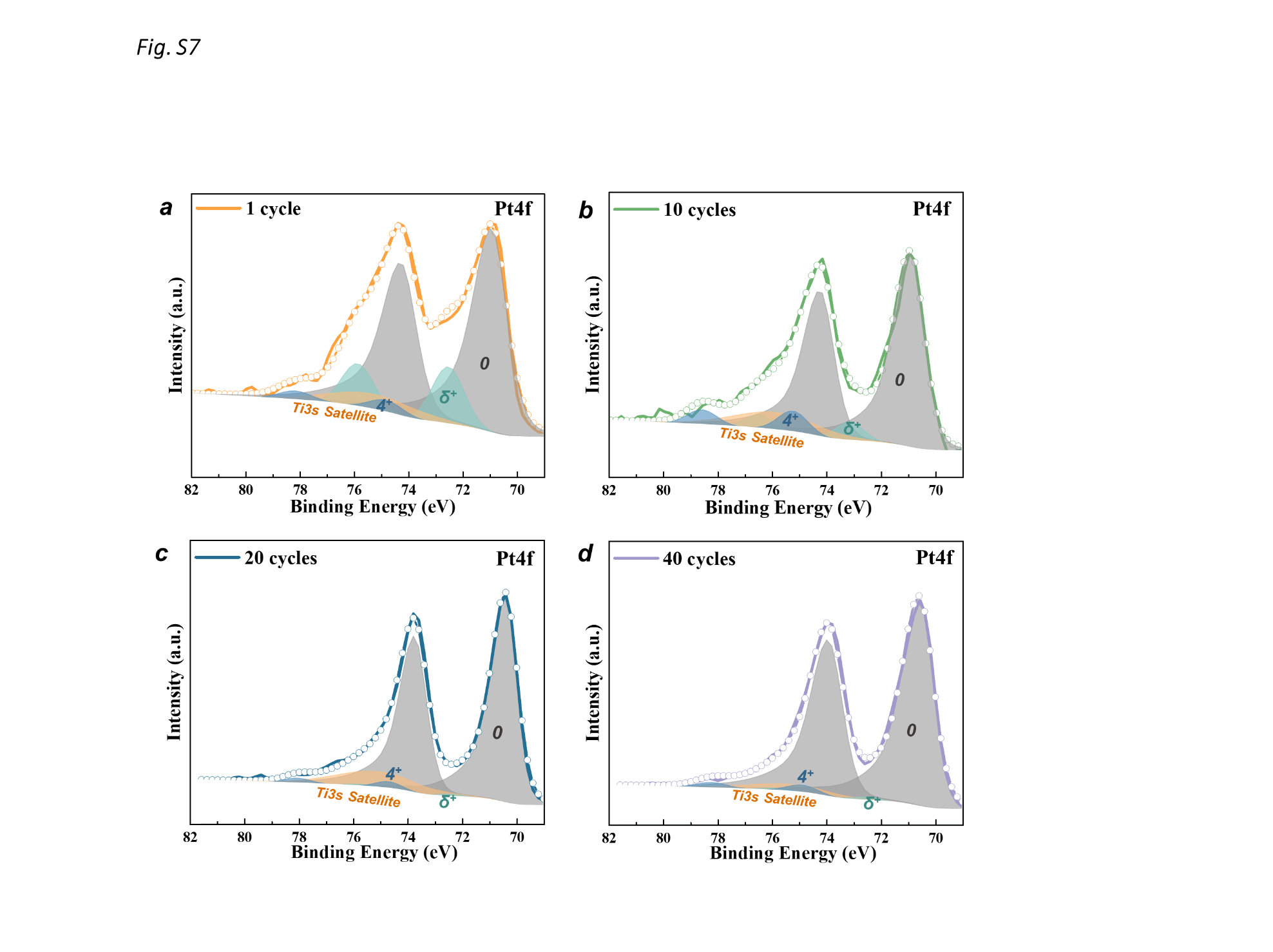


**Figure S10.** Deconvoluted XPS Pt4f spectra of Pt loaded TiO_2_ layers after different cycles.


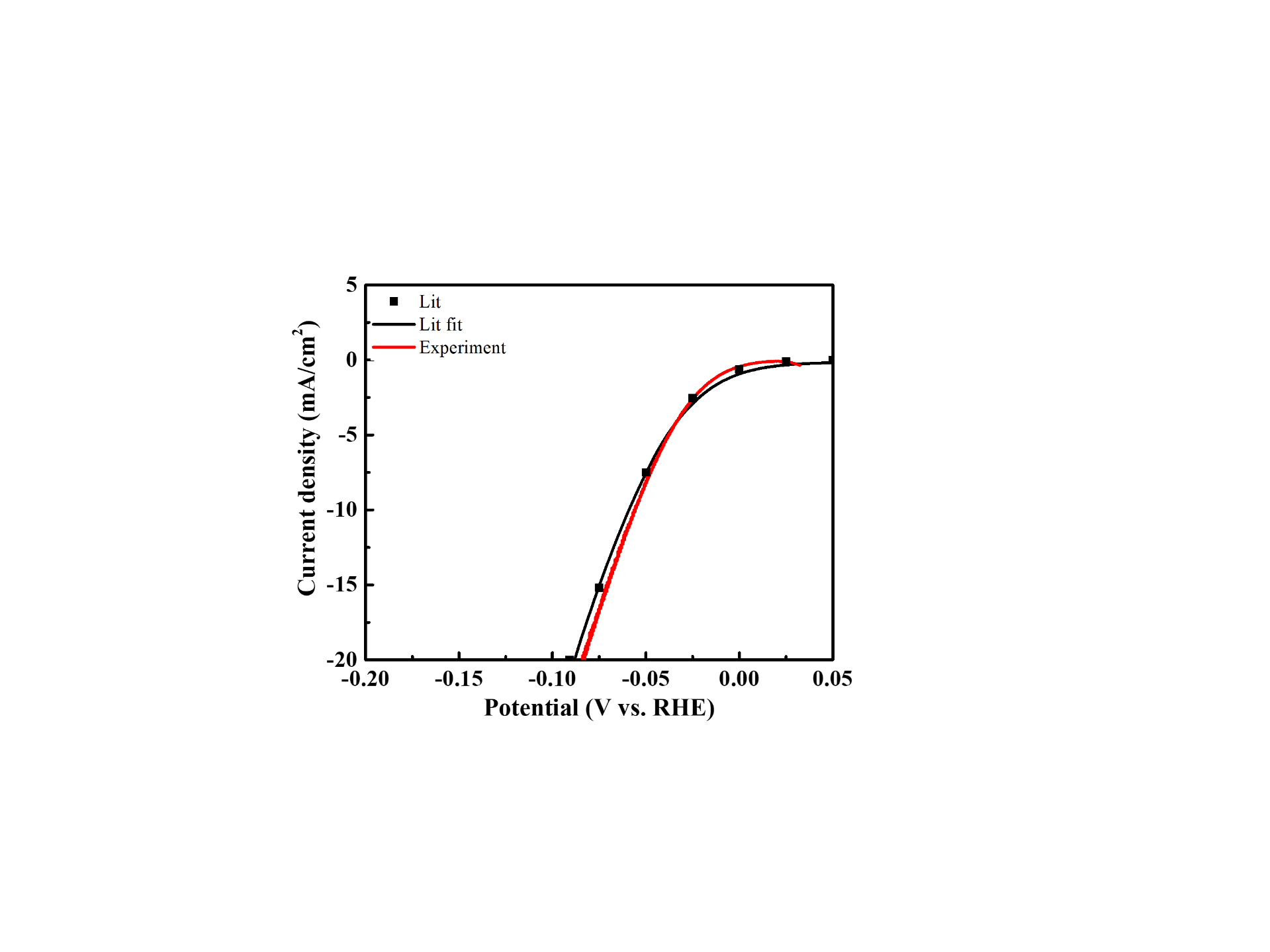


**Figure S11.** IV curves for the HER on Pt foil (0.5 M H_2_SO_4_, 0.5 mV/s scan rate, 1 cm² working electrode area, 0.5 cm working electrode-reference electrode distance).

To compare our data to literature approaches, we followed a most established protocol, ensuring that all crucial parameters for acquiring IV curves on Pt of the HER (0.5 M H_2_SO_4_, 0.5 mV/s scan rate, 1 cm² working electrode area, 0.5 cm working electrode-reference electrode distance, H_2_ purging) matched our experiment^[2]^. Under these conditions, our results are comparable to the literature. Specifically, we measured Pt foil data under identical conditions as a reference to demonstrate the good activity of our samples.

**Table S1** Pt loading on TiO_2_ layers from XPS and conversion to Pt single atom density

| Precursor concentration | **Pt loading (At. %)** | **Pt SA surface density** |
| --- | --- | --- |
| 0.5 mM | **1.67** | 5.5 × 10^6^/µm^2^ |
| 0.1 mM | **0.79** | 2.2 × 10^6^/µm^2^ |
| 0.09 mM | **0.75** | 2.1 × 10^6^/µm^2^ |
| 0.08 mM | **0.56** | 1.6 × 10^6^/µm^2^ |
| 0.07 mM | **0.52** | 1.5 × 10^6^/µm^2^ |
| 0.06 mM | **0.38** | 1.1 × 10^6^/µm^2^ |
| 0.05 mM | **0.34** | 9.6 × 10^5^/µm^2^ |
| 0.04 mM | **0.26** | 7.4 × 10^5^/µm^2^ |
| 0.03 mM | **0.19** | 5.4 × 10^5^/µm^2^ |
| 0.02 mM | **0.17** | 4.7 × 10^5^/µm^2^ |
| 0.01 mM | **0.10** | 2.8 × 10^5^/µm^2^ |
| 0.005 mM | **0.08** | 2.2 × 10^5^/µm^2^ |
| 0.001 mM | **0.01** | 2.9 × 10^4^/µm^2^ |

Note:

The reactive deposition uses H_2_PtCl_6_ solution where the Pt${Cl}_{6}^{2-}$ complex hydrolyzes in the water to a full range of Pt^4+^Cl_x_(H_2_O)_y_(OH)_z_ complexes^[3]^. The speciation depends strongly on the solution concentration (namely: Cl content and pH). I.e. when using H_2_PtCl_6_ of different concentrations not only the Pt content, the Cl content but also the pH is changed. This hydrolysis effects lead to non-linearly correlated deposition amounts. Further details can be found in references [Ref. 24, 43]^[3]–[6]^.

**Table S2** Area ratios for the different Pt species (based on XPS) of Pt loaded TiO_2_ layers after different potential polarization

|  | **0** | **δ^+^** | **4^+^** |
| --- | --- | --- | --- |
| OCP | 0% | 73.27% | 26.73% |
| -0.7V | 4.99% | 74.34% | 20.67% |
| -1.2V | 67.88% | 23.82% | 8.30% |
| -1.5V | 86.89% | 9.46% | 3.65% |

**Table S3** Area ratios for the different Pt species (based on XPS) of Pt loaded TiO_2_ layers after different cycles

|  | **0** | **δ^+^** | **4^+^** |
| --- | --- | --- | --- |
| 1 cycle | 80.45% | 16.00% | 3.55% |
| 10 cycles | 86.89% | 6.03% | 7.08% |
| 20 cycles | 96.76% | 0.83% | 2.41% |
| 40 cycles | 96.36% | 1.02% | 2.62% |

*References*

[1] J.M. Lantz, R.M. Corn, *J. Phys. Chem.* **1994**, *98*, 4899.

[2] Q. Yu, Z. Zhang, H. Liu, X. Kang, S. Ge, S. Li, L. Gan, B. Liu, *Fundam. Res.* **2023**, *3*, 804.

[3] J.R. Regalbuto, A. Navada, S. Shadid, M.L. Bricker, Q. Chen, *J. Catal.* **1999**, *184*, 335.

[4] W. Spieker, J. Regalbuto, D. Rende, M. Bricker, Q. Chen, in *Stud. Surf. Sci. Catal.*, Elsevier**2000**, 203.

[5] W.A. Spieker, J.R. Regalbuto, *Chem. Eng. Sci.* **2001**, *56*, 3491.

[6] X. Hao, W.A. Spieker, J.R. Regalbuto, *J. Colloid Interface Sci.* **2003**, *267*, 259.
